# Exogenous salicylic acid treatment enhances the disease resistance of *Panax vietnamensis* by regulating secondary metabolite production

**DOI:** 10.3389/fpls.2024.1428272

**Published:** 2024-08-16

**Authors:** Jiae Hou, Mingtao Ai, Jianbin Li, Xiuming Cui, Yuan Liu, Qian Yang

**Affiliations:** ^1^ Faculty of Life Science and Technology, Kunming University of Science and Technology, Kunming, China; ^2^ Key Laboratory of Panax notoginseng Resources Sustainable Development and Utilization of State Administration of Traditional Chinese Medicine, Kunming, China; ^3^ Yunnan Provincial Key Laboratory of Panax notoginseng , Kunming, China; ^4^ Kunming Key Laboratory of Sustainable Development and Utilization of Famous-Region Drug, Kunming, China; ^5^ Sanqi Research Institute of Yunnan Province, Kunming, China

**Keywords:** salicylic acid, *Panax vietnamensis*, flavonoid metabolism, lignin synthesis, plant defense

## Abstract

**Introduction:**

Salicylic acid (SA) is a phenolic compound widely found in plants. It plays a key role in exerting plant disease resistance. *Panax vietnamensis* Ha & Grushv., a valuable medicinal plant, contains high levels of phenolic compounds, which contribute significantly to the resilience of the plant against stress. However, the precise role of SA in regulating the synthesis of secondary metabolites in *P.vietnamensis* remains elusive.

**Methods:**

Two-year-old *P. vietnamensis* seedlings were treated with exogenous SA. We systematically assessed the changes in the physiological parameters of SA-treated *P. vietnamensis* leaves, employing transcriptome and metabolome analyses to elucidate the underlying mechanisms.

**Results:**

Our results revealed a significant improvement of the plant’s antioxidant capacity at 6 h post-treatment. Furthermore, exogenous SA treatment promoted the biosynthesis of lignin and flavonoids such as rutin, coumarin, and cyanidin. In addition, it increased the levels of endogenous SA and jasmonic acid (JA), promoting the disease resistance of the plants. Thus, SA pretreatment enhanced the defense of P. vietnamensis against pathogens.

**Conclusions:**

Our study provided novel insights into the potential molecular mechanisms underlying SA-mediated biosynthesis of secondary metabolites. Furthermore, our results provided a theoretical foundation for optimizing the cultivation practices of *P.vietnamensis* and the application of SA as a plant immunomodulator.

## Introduction

Plant growth and development is easily affected by adverse environmental conditions, such as global climate change, environmental pollution, etc. Plant hormones play an important role in mediating plant immune responses against different pathogens (Cao et al., 2022). Plants have evolved several defense mechanisms to resist pathogen attack, including the well-known pathogen-associated molecular patterns (PAMP)-triggered immunity (PTI) (Li et al., 2005), also known as basal resistance (Niks and Marcel, 2009). PTI is mediated by pattern-recognition receptors (PRRs) and enhances plant immunity against various pathogens. In addition, plants also exhibit effector-triggered immunity (ETI) (Chisholm et al., 2006), which relies on host resistance proteins to induce a longer-lasting and more robust immune response. Pathogen invasion can cause rapid local necrosis and cell death, referred to as the hypersensitive response (HR), which is one of the visible manifestations of ETI.

Salicylic acid (SA) is an indispensable hormone involved in plant growth and development (Rivas-San Vicente and Plasencia, 2011). In addition, it plays an important role in plant responses to environmental stresses. Exogenous application of SA facilitates seed germination, growth, and flowering, upregulates photosynthesis, and enhances the activities of enzymes and non-enzymatic antioxidants (Arif et al., 2020). Moreover, SA plays a key role in stomatal closure and senescence (Morris et al., 2000; Metwally et al., 2003; Rajjou et al., 2006). Furthermore, it is known to alleviate plant abiotic stresses, such as heat, cold, salt, heavy metals, drought, ultraviolet radiation, and other stresses (Liu et al., 2022). SA regulates plant growth and environmental stress response by altering endogenous SA levels and the expression of the related downstream genes. Plants are known to fight against pathogen infection by accumulating SA. Non-expressor of pathogenesis-related genes (NPR) proteins play a pivotal role in plant immunity. These proteins serve as important transcription co-activators within the SA signal transduction pathway, thus working in conjunction with SA to enhance plant disease resistance (Hönig et al., 2023).

Plant hormones play an important role in all aspects of plant growth, including the biosynthesis of plant secondary metabolites, such as flavonoids (Coelho et al., 2019). SA, a crucial signaling molecule in plants, participates in a wide range of biochemical and biomolecular processes. These processes can influence plant growth and development and the production of secondary metabolites (Khan et al., 2015). Previous studies have shown that exogenous SA treatment can promote the accumulation of total phenols in chickpea (War et al., 2011) and phenolic compounds in *Salvia miltiorrhiza* cells (Dong et al., 2010). In addition, exogenous SA application can also increase the levels of flavonoids, including caffeic acid, rosmarinic acid, rutin, and anthocyanin, across various plant species, such as *Ginkgo biloba*, *Carthamus*, *Capsicum*, zinger, and more (Ali, 2021; Ejtahed et al., 2015; Ni et al., 2018; Ghasemzadeh and Jaafar, 2012).

*Panax vietnamensis* Ha and Grushv., also known as Nanqi, is a member of the *Araliacecae* plant family. This plant was discovered in 1973 in the Yuling Mountain, located in the Kunsong Province, central Vietnam (Dung and Grushvitski, 1985). Contemporary research on *P. vietnamensis* has demonstrated several medicinal properties in this plant. It has been shown to possess anti-inflammatory (Le et al., 2018), anti-tumor (Chirikova et al., 2019), melanin formation inhibition (Le et al., 2014), and hepatoprotective properties (Le et al., 2023). Due to the scarcity of wild germplasm resources of *P. vietnamensis* and its high medicinal value, people have over-exploited this plant, severely damaging its ecosystem. Although large-scale artificial planting effectively protects and utilizes *P. vietnamensis*, this approach exposes plants to various diseases, resulting in the frequent use of fungicides. This practice severely affects the yield and quality of *P. vietnamensis*. Moreover, to the best of our knowledge, there have been no reports on *P. vietnamensis* exhibiting disease resistance.

SA, as a key hormone involved in plant defense, plays an important role in plant disease resistance. This study aimed to determine the effect of SA on stress resistance in *P. vietnamensis* and whether SA affects the secondary metabolism in *P. vietnamensis*. We used transcriptomic and metabolomic techniques to analyze the influence of exogenous SA on the growth and development of *P. vietnamensis*. We explored the metabolic pathways of phenols (flavonoid and lignin syntheses) and the SA and JA biosynthesis pathways. This study provided a detailed transcriptomic and metabolomic framework of SA-treated *P. vietnamensis* leaves. Our results provided valuable insights into the disease resistance of *P. vietnamensis* after SA application and a theoretical foundation for the commercialization of SA as a plant immunomodulator for *P. vietnamensis*.

## Materials and methods

### Plant materials and growth conditions

Two-year-old *P. vietnamensis* plants were used for this study. The plants were obtained from the *P. vietnamensis* planting base in Yuanyang County, Yunnan Province, China (N 22°53’50”, E 103°20’8”; altitude: 1691.54 m) and grown in the greenhouse of Kunming University of Science and Technology (N 24°51’27”, E 102°51’12”; altitude: 1913.67 m). After acclimatization for 2 weeks (temperature: 20 ± 2 °C, humidity: 70%, 16-h light/8-h dark photoperiod), the plants were treated with SA.

### SA treatment

We sprayed 5 mM SA with 0.005% *v/v* Silwet L-77 onto *P. vietnamensis* leaf surface until it was completely wet. Control (CK) *P. vietnamensis* leaves were sprayed with an equal amount of SA-free solution containing only 0.005% *v/v* Silwet L-77.

The leaves were collected both before (0 h) and after SA treatment (3, 6, 12, and 24 h post-treatment). After quick freezing with liquid nitrogen, the leaves were stored at −80 °C until further use. For transcriptome sequencing and metabonomic analysis, three and six independent biological repeats were collected from each treatment group (CK/SA-0h, CK-6h, SA-6h, CK-24h, SA-24h).

### Measurement of physiological indexes in SA-treated *P. vietnamensis* leaves

The superoxide dismutase (SOD), peroxidase (POD), catalase (CAT), phenylalanine ammonia-lyase (PAL), and polyphenol oxidase (PPO) levels in exogenous SA-treated *P. vietnamensis* leaves were assayed using respective kits (Bioengineering, Nanjing, China) according to the manufacturer’s protocols. The protein levels in *P. vietnamensis* leaves were determined using a bicinchoninic acid (BCA) protein assay kit (Solarbio, China). Three biological replicates were used for each analysis.

### Disease resistance assays

*Neofusicoccum ribis* was cultivated on solid potato dextrose agar (PDA) culture medium (comprising 200 g potato, 20 g glucose, and 15 g agar) at 28°C in the dark for one week. *N. ribis* was inoculated on *P. vietnamensis* leaves pretreated with SA for 6 h and 24 h. Then, the inoculated plants were cultured in the greenhouse under a 16-h light/8-h dark photoperiod at temperatures of 25°C (day) and 20°C (night). The size of diseased spots on the inoculated leaves was observed after four days.

### Analysis of transcriptome and metabolome

Method S1 details the protocols for RNA extraction, library preparation, sequence analysis, and bioinformatics analysis used for transcriptome analyses. Method S2 details the protocols for metabolite extraction, detection, identification, and quantification and differential metabolite statistics for metabolome analyses.

### Correlation analysis of transcriptomic and metabolomic data

Pearson correlations were calculated for the correlation analysis between the differentially expressed genes (DEGs) and the various metabolites. Correlations with coefficient (r) value > 0.5 or < −0.5 and P < 0.05 were considered as the crucial relationships between the transcriptome and the metabolome. In order to visualize the specific relationships, a correlation network analysis was performed using the Cytoscape software (v3.9.0).

### Quantitative real-time polymerase chain reaction

Total RNA was extracted from *P. vietnamensis* leaves using Trizol reagent (Takara). One microgram of RNA sample was reverse transcribed using a Prime Script RT reagent Kit (with gDNA Eraser, Takara). All primers used for qRT-PCR are listed in **Supplementary Table S1**. *PvACTIN7* was used as the internal reference gene. Relative gene expression level was calculated using the 2^−ΔΔCT^ method (Livak and Schmittgen, 2001).

### Determination of lignin content using high-performance liquid chromatography-mass spectrometry

The dried leaves of *P. vietnamensis* were ground into powder, and 0.1 g of the powdered sample was hydrolyzed with 4 mol/L NaOH solution at 95°C for 24 h. After cooling, 6 mol/L HCl was added to the mixture for neutralization. The samples were centrifuged at 13,000 rpm for 5 min. Then, 500 µL of supernatant was extracted twice with 1 mL of ethyl acetate. The organic phase was collected, blown dry with nitrogen, redissolved in ultrapure water, and passed through a 0.22 µm filter membrane. Lignin concentrations were analyzed using HPLC-MS as described previously (Yang et al., 2022).

### Determination of endogenous SA and jasmonic acid concentrations using HPLC-tandem mass spectrometry

P.*vietnamensis* leaves (3 g) were ground in liquid nitrogen and placed in a test tube. The powder was mixed with isopropanol-water-hydrochloric acid solution and 8 µL of 1 µg/mL tritium internal standard solution. The mixture was shaken at 4°C for 30 min. Next, dichloromethane was added to the mixture, and it was shaken again at 4°C for 30 min. Then, the mixture was centrifuged at 4°C at 13,000 rpm for 5 min. The organic phase was collected in the dark, blow-dried with nitrogen, and redissolved in methanol (containing 0.1% formic acid). The samples were then filtered through a 0.22 μm membrane and analyzed using HPLC-MS/MS. Using methanol (0.1% formic acid) as the solvent, SA and JA standard solutions with varying gradients (0.1, 0.2, 0.5, 2, 5, 20, 50, and 200 ng/mL) were prepared. For HPLC, mobile phase A comprised methanol/0.1% formic acid, and mobile phase B comprised water/0.1% formic acid. A Poroshell 120 SB-C18 reversed-phase chromatographic column (2.1 mm × 150 mm, 2.7 μm diameter) was used to separate SA and JA. The HPLC gradient program was as follows: 0–1 min, A = 20%; 1–9 min, A increased to 80%; 9–10 min, A = 80%; 10–10.1 min, A decreased to 20%; 10.1–15 min, A = 20%. The sample volume and column temperature were 2 μL and 30°C, respectively.

### Cyanidin assessment using HPLC-MS/MS

*P. vietnamensis* leaves (1 g) were ground in liquid nitrogen. The powder was mixed with 15 mL of ethanol/HCl extraction buffer and subjected to ultrasound sonication (40 kHz) for 30 min. The mixture was centrifuged at 4°C and 13,000 rpm for 5 min. The supernatant was collected, and the residue was re-extracted once. The two extracts were combined, mixed with 7 mL HCl, and incubated in a water bath at 95°C for 40 min. Then, the mixture was cooled down to room temperature, filtered through a 0.22-μm membrane, and analyzed using HPLC-MS/MS. Using methanol (containing 0.1% formic acid) as the solvent, the cyanidin standard solutions of varying gradients of (10, 20, 50, and 100 μg/mL) were prepared. For HPLC, mobile phase A comprised 0.1% formic acid/water, and mobile phase B comprised 0.1% formic acid/acetonitrile. A Nova Atom C18 column (4.6 mm × 250 mm) was used for HPLC. The HPLC gradient program was as follows: 0–2 min, B = 8%; 2–5 min, B increased to 12%; 5–10 min, B = 18%; 10–12 min, B increased to 25%; 12–15 min, B increased to 30%; 15–18 min, B increased to 45%; 18–20 min, B decreased to 20%; 20–22 min, B = 8%. The sample volume and column temperature were 10 μL and 30°C, respectively.

### Determination of coumarin and rutin by ultra-high-performance liquid chromatography-MS/MS

P. *vietnamensis* leaves (1 g) were ground in liquid nitrogen. Then, 0.5 g of the powder was weighed and added to a 50-mL test tube. Next, 10 mL of 80% methanol was added to the sample, and the mixture was subjected to ultrasound sonication (40 kHz) at 23°C for 50 min. The mixture was then centrifuged at 4°C for 10 min at 4000 g. The supernatant was blow-dried with nitrogen and redissolved in 80% methanol. The samples were filtered through a 0.22-μm membrane, and the filtrate was analyzed using UPLC-MS as described previously (Zhao and Shi, 2022).

### Data analysis

Excel software was used to sort out the statistical test data. Prism8.0.1 software (GraphPad software), IBM SPSS Statistics 22.0 software, and Cytoscape software (v3.9.0) were used to analyze the related data. The Duncan method was used for multiple comparisons (^*^P < 0.05 or ^**^P < 0.01).

## Results

### Oxidative stress response of SA-treated *P. vietnamensis* leaves

**Figure 1:**
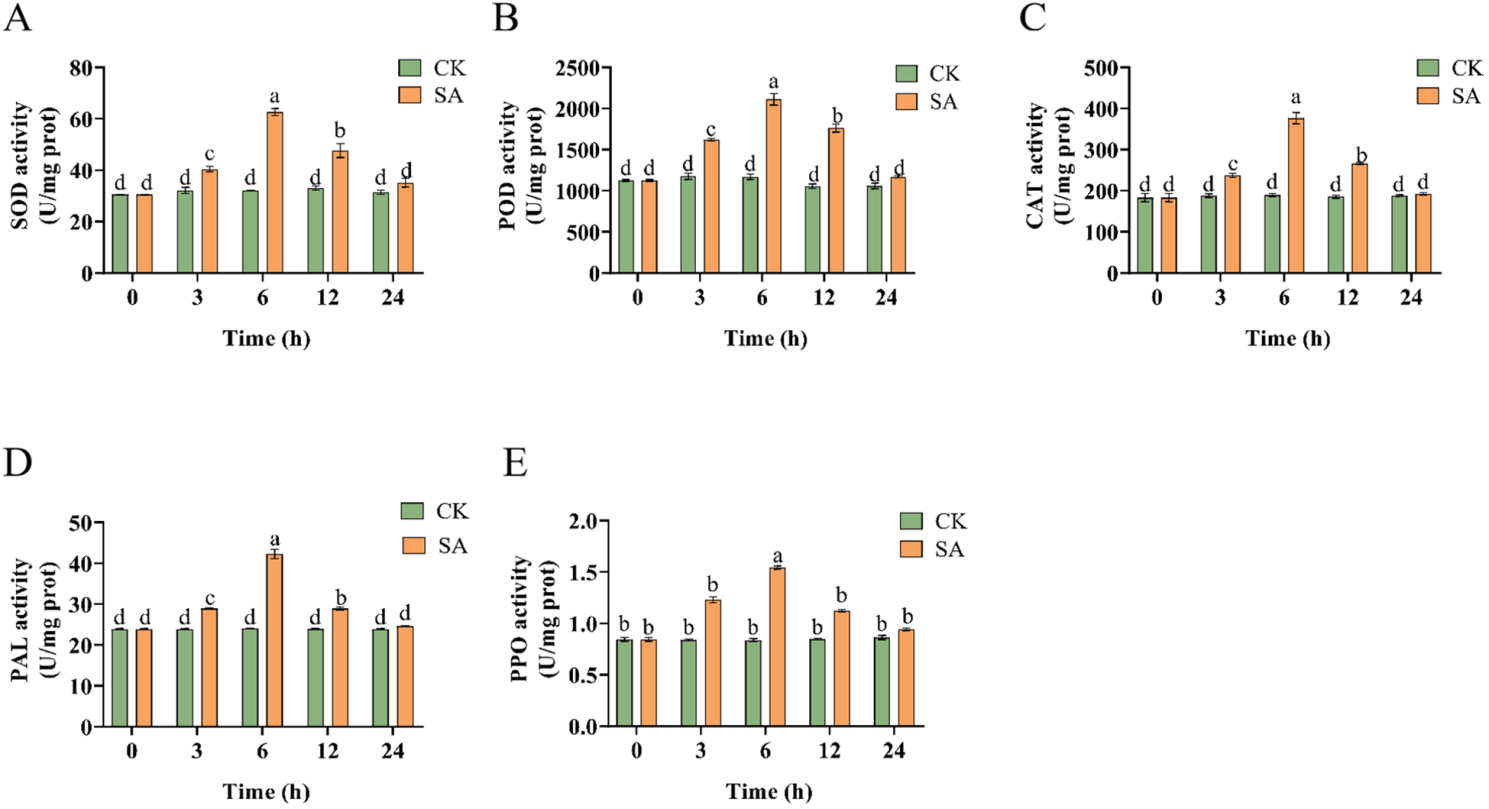
Changes of enzyme activities of *P. vietnamensis* leaves during SA treatment. **(A)** SOD activity (u/mg prot), **(B)** POD activity (U/mg prot), **(C)** CAT activity (U/mg prot), **(D)** PAL activity (U/mg prot), **(E)** PPO activity (U/mg prot). The values are the means ± SEs (n = 3). Different letters indicate significant differences at P < 0.05 (Student’s t-test).

We detected the activity levels of SOD, POD, CAT, PAL, and PPO in SA-treated *P. vietnamensis* leaves to evaluate the antioxidant enzyme activity of *P. vietnamensis* plants post-SA treatment. The results showed that the activities of these enzymes peaked at 6 h post-SA treatment (**Figure 1**), with a significant difference between the activity levels at 6 h and those at other time points. This finding showed that antioxidant enzymes participated in the scavenging of reactive oxygen species (ROS) and the synthesis of immunity-related substances, such as phenols, lignin, and phytochemicals. These substances also effectively scavenge ROS, thus protecting biological macromolecules and cell membranes from oxidative damage and strengthening the disease-resistance ability of *P. vietnamensis.*

### Transcriptome analysis of SA-treated *P. vietnamensis*

**Figure 2:**
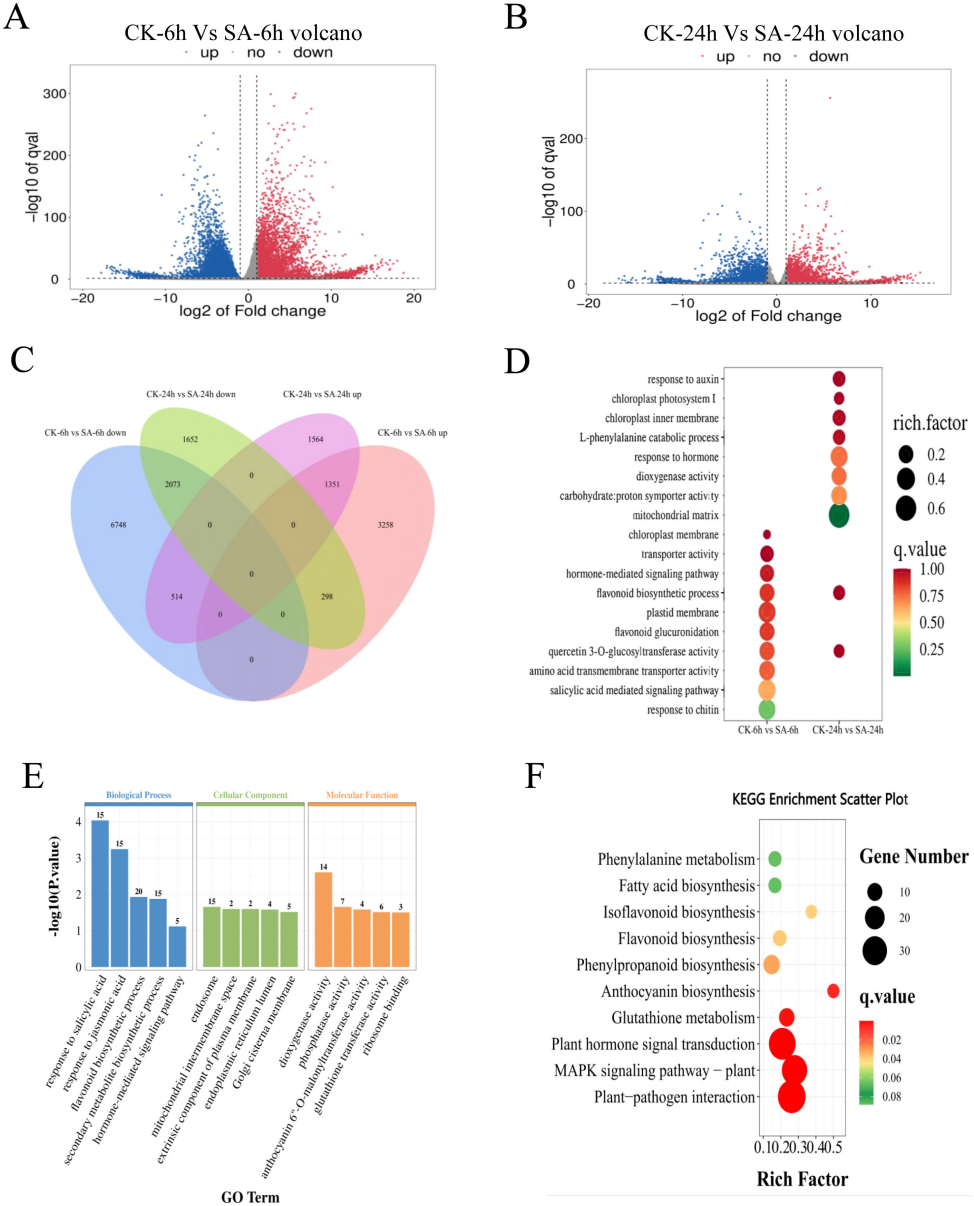
Transcriptome analysis of SA-treated and control *P. vietnamensis* samples. **(A)** Volcanic map of DEGs in *P. vietnamensis* CK-6h vs SA-6h. **(B)** Volcanic map of DEGs in *P. vietnamensis* CK-24h vs SA-24h. **(C)** The venn diagram of DEGs up-regulated and down-regulated of *P. vietnamensis* in CK-6h vs SA-6h and CK-24h vs SA-24h. **(D)** GO analysis of DEGs identified in CK-6h vs SA-6h and CK-24h vs SA-24h. Only some terms were listed (P < 0.05). **(E)** GO analysis was performed on the differentially expressed genes up-regulated in CK-6h vs SA-6h and CK-24h vs SA-24h (P < 0.05). **(F)** KEGG enrichment analysis was performed on the overlapping differentially expressed genes in up-regulated in CK-6h vs SA-6h and CK-24h vs SA-24h (P < 0.05).

The total number of clean reads for samples obtained by high-throughput sequencing ranged from 0.9223 million to 143.81 million. The filtered Q30 of each sample was >95%, and the GC ratio was between 42.5% and 44%, indicating the high quality of RNA-sequencing (RNA-seq) data. More than 78.49% of the clean data can be mapped to *P. vietnamensis* genome, and more than 68.21% of the unique maps were read (**Table 1**). These results indicated that the transcriptome data was sufficiently accurate to further identify differentially expressed genes (DEGs). Fragments Per Kilobase of transcript per Million mapped reads (FPKM) analysis was performed to assess the transcription abundance of the DEGs. The genes with a false discovery rate (FDR) of <0.05 and |log2(FoldChange)| > 1 were screened as DEGs. Furthermore, we compared the DEGs in the SA-6h and SA-24h groups to further elucidate the SA-responsive genes. We detected more DEGs in the SA-6h group than in the SA-24h group (**Figures 2A, B**). More specifically, Venn diagram analysis showed 14242 DEGs in the CK-6h vs SA-6h group, including 4907 upregulated and 9335 downregulated genes. In the CK-24h vs SA-24h group, 7452 DEGs were detected, with 3429 upregulated and 4023 downregulated genes. In CK-6h vs. SA-6h and CK-24h vs. SA-24h, 1351 and 2073 genes were significantly upregulated and downregulated, respectively (**Figure 2C**).

**Table 1:** Quality of the sequencing data.

Samples	total reads	clean reads	GC (%)	Q30 (%)	unique mapped reads	multiple mapped reads	total mapped reads
CK/SA-0 h-1	125,917,114	108,109,466 (85.86%)	44.00%	96.55%	75,742,841 (70.06%)	9,112,416 (8.43%)	84,855,257 (78.49%)
CK/SA-0 h-2	139,806,628	133,260,974 (95.32%)	42.50%	96.25%	101,246,632 (75.98%)	13,190,655 (9.90%)	114,437,287 (85.88%)
CK/SA-0 h-3	113,207,062	111,343,070 (98.35%)	43.50%	95.94%	75,942,537 (68.21%)	11,526,645 (10.35%)	87,469,182 (78.56%)
CK-6 h-1	139,327,908	118,863,010 (85.31%)	44.00%	97.56%	92,179,761 (77.55%)	9,636,623 (8.11%)	101,816,384 (85.66%)
CK-6 h-2	108,258,462	92,232,496 (85.20%)	44.00%	96.97%	73,008,257 (79.16%)	7,337,658 (7.96%)	80,345,915 (87.12%)
CK-6 h-3	128,560,230	106,742,214 (83.03%)	44.00%	96.32%	84,817,201 (79.46%)	8,483,737 (7.95%)	93,300,938 (87.41%)
CK-24 h-1	124,075,592	106,690,468 (85.99%)	44.00%	96.71%	83,520,204 (78.28%)	7,556,169 (7.08%)	91,076,373 (85.36%)
CK-24 h-2	123,908,000	107,481,940 (86.74%)	44.00%	96.72%	79,608,142 (74.07%)	7,449,384 (6.93%)	87,057,526 (81%)
CK-24 h-3	143,039,702	137,014,690 (95.79%)	43.00%	96.50%	107,310,521 (78.32%)	9,839,346 (7.18%)	117,149,867 (85.5%)
SA-6 h-1	121,862,788	103,589,878 (85.01%)	43.00%	96.64%	82,604,614 (79.74%)	8,604,180 (8.31%)	91,208,794 (88.05%)
SA-6 h-2	129,160,874	100,310,112 (77.66%)	43.00%	96.70%	79,824,795 (79.58%)	8,215,356 (8.19%)	88,040,151 (87.77%)
SA-6 h-3	123,573,882	104,499,280 (84.56%)	43.00%	96.75%	83,094,366 (79.52%)	9,193,347 (8.80%)	92,287,713 (88.32%)
SA-24 h-1	147,659,406	141,316,240 (95.70%)	43.00%	96.48%	118,231,335 (83.66%)	9,267,628 (6.56%)	127,498,963 (90.22%)
SA-24 h-2	146,925,168	140,919,472 (95.91%)	43.00%	96.47%	118,004,627 (83.74%)	9,734,678 (6.91%)	127,739,305 (90.65%)
SA-24 h-3	150,572,104	143,810,234 (95.51%)	43.00%	96.34%	121,404,526 (84.42%)	8,925,038 (6.21%)	130,329,564 (90.63%)

GO analysis of CK-6h vs SA-6h and CK-24h vs SA-24h showed that SA induced several functional genes. GO enrichment of DEGs can be divided into three main categories: Biological process (BP), cellular component (CC), and molecular function (MF) (**Figure 2D**). The genes related to “flavonoid biosynthesis” and “quercetin 3-O-glucosyltransferase activity” were enriched in both comparison groups. In the BP class, the CK-6h vs. SA-6h comparison group revealed enrichment of “salicylic acid mediated signal pathway” and “hormone-mediated signaling pathway” terms while the CK-24h vs. SA-24h comparison group showed enrichment of “catabolic process of L- phenylalanine” and “response to auxin” terms. In the CC class, the terms “chloroplast membrane” and “plastid membrane” were enriched in CK-6h vs. SA-6h, while the terms “chloroplast intima,” “mitochondrial matrix,” and “chloroplast photosystem II” were enriched in CK-24h vs. SA-24h. In the MF class, the terms “transporter activity” and “amino acid transmembrane transporter activity” were enriched in CK-6h vs. SA-6h, while the terms “dioxygenase activity” and “carbohydrate:proton symporter activity” were enriched in CK-24h vs. SA-24h. Next, we performed functional classification and enrichment analysis of the DEGs in CK-6h vs. SA-6h and CK-24h vs. SA-24h to elucidate the differences in the functions of the genes at 6 and 24 h post-SA treatment (**Figure 2E**; **Supplementary Figure S1**). The upregulated DEGs enriched in the terms “response to salicylic acid,” “response to jasmonic acid,” “flavonoid biosynthesis process,” “secondary metabolite biosynthetic process,” and “hormone-mediated signaling pathway”. The downregulated DEGs primarily enriched to the terms “photosynthesis” and “photosystem II repair”.

According to KEGG enrichment analysis, the upregulated DEGs in CK-6h vs. SA-6h and CK-24h vs. SA-24h primarily enriched to the terms “plant pathogen interaction,” “MAPK signal pathway-plant,” “plant hormone signal transduction,” “anthocyanin biosynthesis,” “phenylpropanoid biosynthesis,” “flavonoid biosynthesis” (P value < 0.05) (**Figure 2F**). The downregulated DEGs in CK-6h vs. SA-6h and CK-24h vs. SA-24h enriched to the terms “photosynthesis,” “photosynthesis - antenna proteins,” and “circadian rhythm - plant” (**Supplementary Figure S2**). These results further indicated that exogenous SA elicits disease resistance-associated defense responses and induction of secondary metabolic pathway-related genes at transcriptional level.

### qRT-PCR to validate RNA-seq data

**Figure 3:**
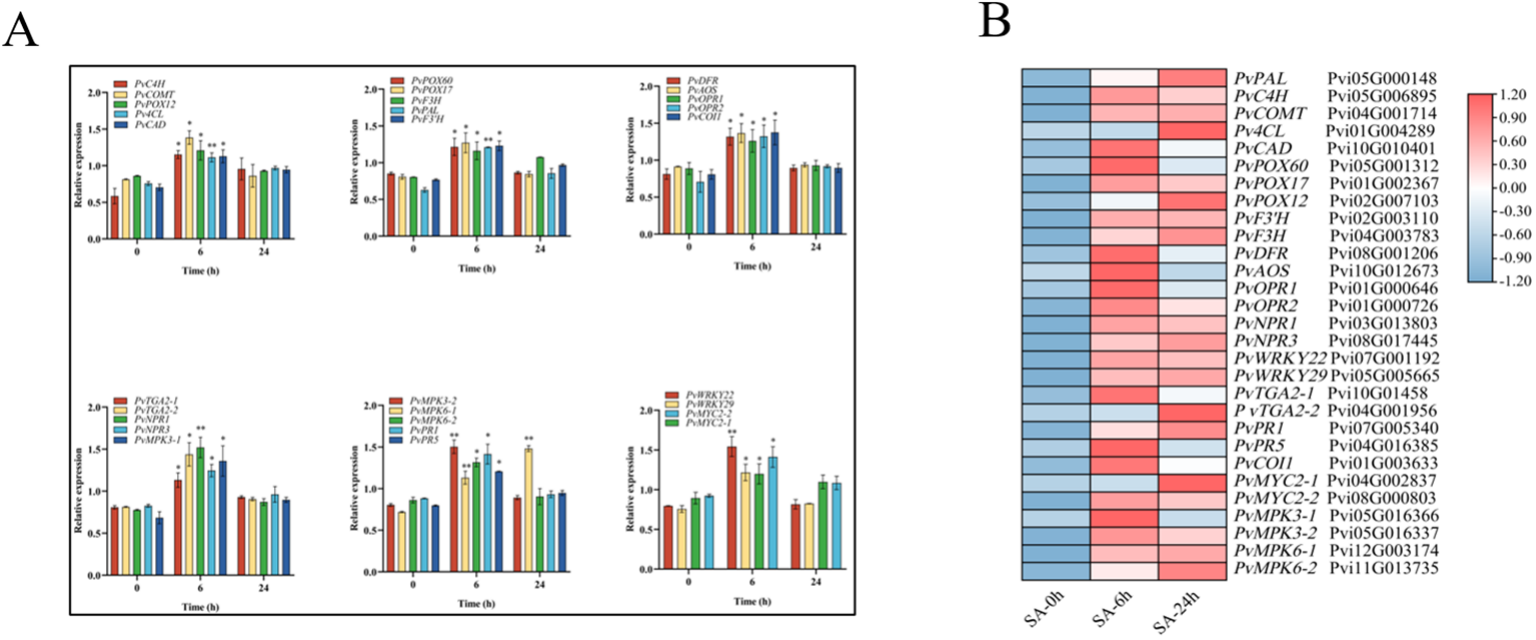
Analysis of expression level of 29 DEGs after SA treatment (CK/SA-0h, SA-6h, SA-24h). **(A)** qRT-PCR. **(B)** RNA-seq data. The transcriptional levels were assessed using FPKM values based on transcriptomic data. Expression data were gene-normalized by the expression data using Tbtools software. The Value -1.2 to 1.2 was defaultly set with the color scale limits according to the normalized value. The color scale shows increasing expression levels from blue to red. Asterisks indicate the significant level (n = 3, *P < 0.05, **P < 0.01) based on a Tukey’s honestly significant difference test.

Next, we randomly selected 29 DEGs and subjected them to qRT-PCR to validate the accuracy of the RNA-seq data (**Figure 3A**). Our results showed that the SA-6h group exhibited an upregulation of the genes related to lignin synthesis (*PvPAL*, *PvC4H*, *PvCOMT*, *Pv4CL*, *PvCAD*, *PvPOX60*, *PvPOX17*, and *PvPOX12*), flavonoid synthesis (*PvF3H*, *PvF3’H*, and *PvDFR*), SA synthesis (*PvPAL*, *PvTGA2-1*, and *PvTGA2-2*), JA synthesis (*PvAOS*, *PvOPR1*, *PvOPR2*, and *PvCOI1*), and disease resistance and immunity (*PvNPR1*, *PvNPR3*, *PvMPK3-1*, *PvMPK3-2*, *PvMPK6-1*, *PvMPK6-2*, *PvPR1*, and *PvPR5*). At the same time, the FPKM values (SA-0h, SA-6h, and SA-24h) showed that the expression levels of genes related to lignin, flavonoids, SA and JA, and disease resistance and immunity were similar to those observed via qRT-PCR (**Figure 3B**).

### Metabolomic analysis of SA-treated *P. vietnamensis*

**Figure 4:**
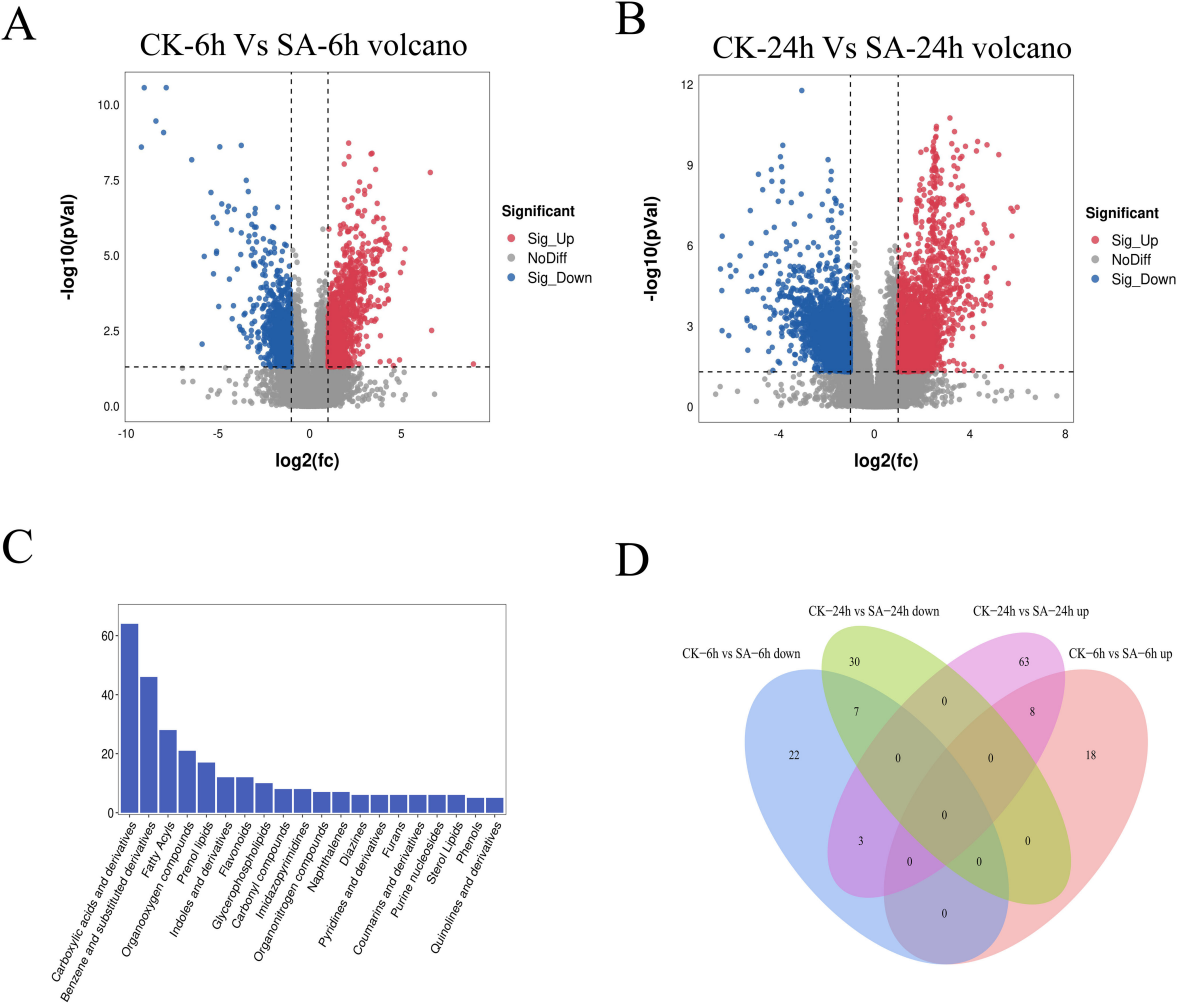
Metabonomics analysis of *P. vietnamensis* treated with SA for 6h and 24h. **(A)** Volcanic diagram of differential metabolites in *P. vietnamensis* CK-6h vs SA-6h. **(B)** Volcanic diagram of differential metabolites in *P. vietnamensis* CK-24h vs SA-24h. **(C)** Classification of detected metabolites. **(D)** Venn diagram analysis of significantly different metabolites *P. vietnamensis* in CK-6h vs SA-6h and CK-24h vs SA-24h.

Next, we performed differential metabolite analysis to understand the effects of exogenous SA application on the metabolites in *P. vietnamensis* leaves. Our results showed significantly more SA-responsive metabolites in the SA-24h group than in the SA-6h group (**Figures 4A, B**). We detected 286 metabolites, divided into 20 categories, primarily including carboxylic acids and their derivatives, flavonoids, glycerophosphates, coumarins, and phenols (**Figure 4C**). In the SA-6h group, 58 metabolites were identified, with 26 upregulated and 32 downregulated metabolites. In the SA-24h group, 111 metabolites were identified, with 74 upregulated and 37 downregulated metabolites (**Supplementary Figure S3**). Further analysis revealed 18 differential metabolites between SA-6h and SA-24h groups (**Figure 4D**; **Supplementary Table S2**). In CK-6h vs. SA-6h and CK-24h vs. SA-24h, eight metabolites, including SA, phenol, etc., were upregulated, and seven metabolites, including trigoforin, o-acetyl-L-carnitine, were downregulated. These results indicated that these differential metabolites might be involved in the stress resistance mechanism of *P. vietnamensis* plants.

### Response of lignin and flavonoid metabolic pathways to SA

**Figure 5:**
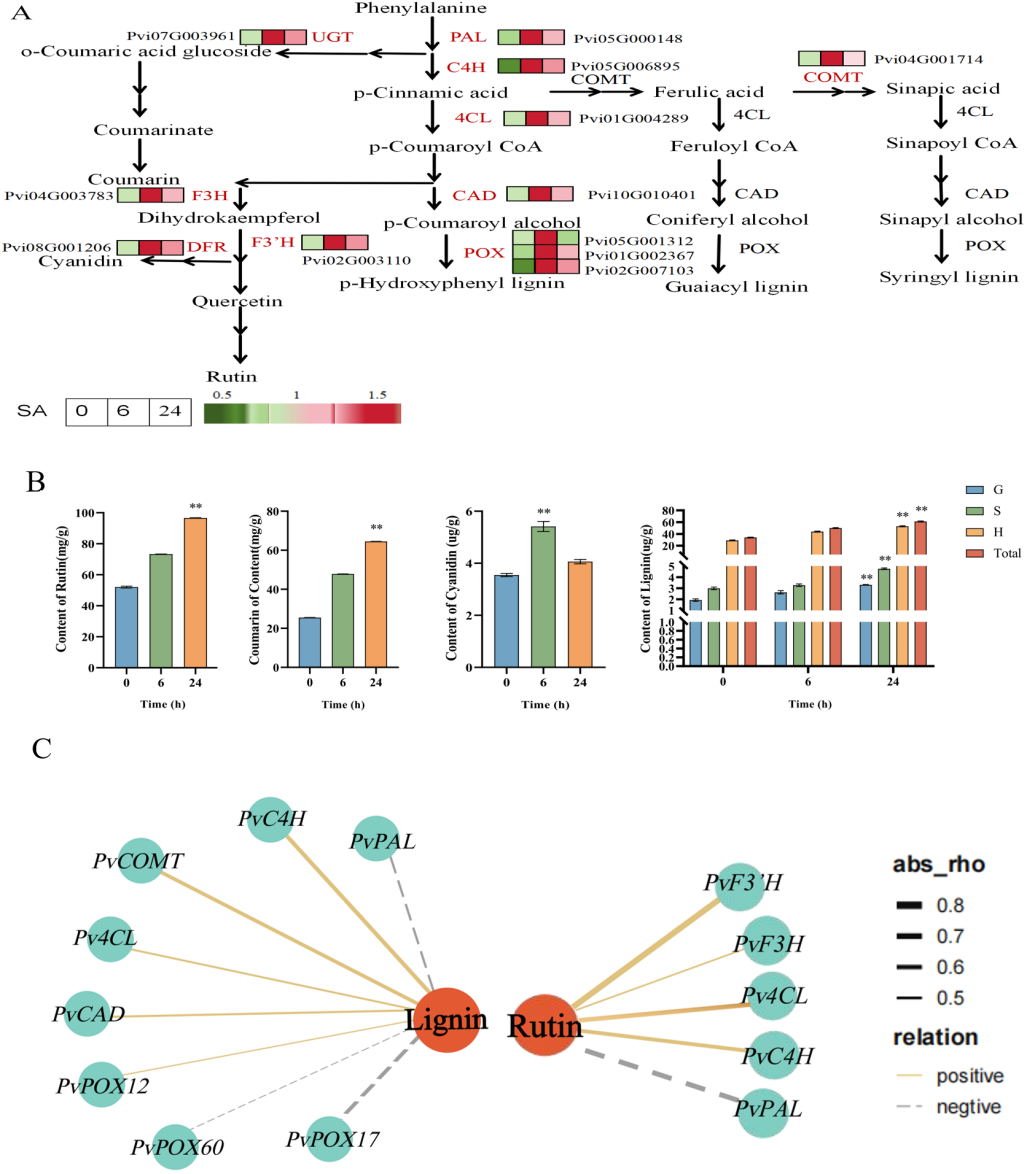
Gene expression pattern and metabolite content analysis of “lignin, coumarin and flavonoid synthesis “ pathway of *P. vietnamensis* under SA treatment. **(A)** Biosynthetic pathway of lignin, coumarin, rutin and cyanidin. The expression of DEGs was expressed by thermogram. Red and green indicate high and low abundance respectively. PAL, phenylalanine ammonia-lyase; C4H, cinnamic acid 4- hydroxylase; 4CL,4- coumaric acid coenzyme A ligase; COMT, caffeic acid -0- methyltransferase; CAD, cinnamyl alcohol dehydrogenase; POX, peroxidase; F3H, flavone 3- hydroxylase; F3’H, flavone 3’- hydroxylase; DFR, dihydroflavonol 4 reductase; UGT,UDP- glycosyltransferase. **(B)** The contents of rutin, coumarin, cortisone and lignin in *P. vietnamensis* were determined after SA treatment. G, S and H represent guaiac lignin, clove lignin and p-hydroxylignin respectively. All the data are the average values of the three biological repeats, and the error bars represent the standard deviations of the three repeats. The asterisk indicated significant level (P < 0.05), and Tukey’s significant difference test was used. **(C)** The interaction network of DEGs and DEMs involved in lignin and rutin biosynthesis. The solid line indicates positive correlation, the dotted line indicates negative correlation, the orange circle indicates DAM, the blue quadrilateral indicates DEGs, and the thick and thin line indicates the strength of correlation.

Polyphenols such as lignin and flavonoids can enhance disease resistance in plants. KEGG enrichment analysis showed that flavonoid, isoflavone, and anthocyanin biosynthesis pathways were significantly enriched in SA-0h vs. SA-6h and SA-0h vs. SA-24h. In addition, flavonoid, flavonol, and isoflavone biosynthesis pathways were significantly enriched in SA-6h vs. SA-24h (**Supplementary Table S3**). The expressions of genes related to phenylalanine, tyrosine, tryptophan, and anthocyanin biosynthesis pathways were upregulated in SA-0h vs. SA-24h (**Supplementary Table S4**). These results showed that exogenous SA significantly induced the biosynthesis of secondary metabolites, such as phenylpropanoid, flavonoids, and lignin. Furthermore, we found that eight genes (*PvPAL*, *PvC4H*, *Pv4CL*, *PvCAD*, *PvCOMT*, *PvPOX60*, *PvPOX17*, and *PvPOX12*) were highly related to lignin biosynthesis. Three genes (*PvF3H*, *PvF3’H*, and *PvDFR*) are highly related to flavonoid biosynthesis. Moreover, most genes upstream of the “lignin, rutin, cyanidin, and coumarin” biosynthesis pathway were also upregulated (**Figure 5A**).

While detecting the contents of lignin and flavonoids in *P. vietnamensis*, a high cyanidin accumulation in the SA-6h group, while the SA-24h group exhibited a high accumulation of lignin, rutin, and coumarin (**Figure 5B**). Furthermore, the co-expression network analysis of the relationship between metabolites and their related synthetic genes showed that lignin positively correlated with *PvCOMT* (Pvi04G001714), which encodes caffeic acid-0-methyltransferase; *PvC4H* (Pvi05G006895), which encodes cinnamic acid 4-hydroxylase; *Pv4CL* (Pvi01G004289), which encodes 4-coumaric acid CoA ligase; *PvCAD* (Pvi10G010401), which encodes cinnamyl alcohol dehydrogenase; and *PvPOX12* (Pvi02G007103), which encodes peroxidase (**Figure 5C**). Rutin positively correlated with *Pv4CL* (Pvi01G004289), which encodes 4-coumaric acid CoA ligase; *PvF3H* (Pvi04G003783), which encodes flavonoid 3’-hydroxylase; and *PvF3’H* (Pvi02G003110), which encodes flavonoid 3’-hydroxylase. These results indicated that the SA hormones act in conjunction with lignin and flavonoids to enhance the defense and disease resistance of plants.

### Effects of SA on the metabolic pathways of endogenous SA and JA

**Figure 6:**
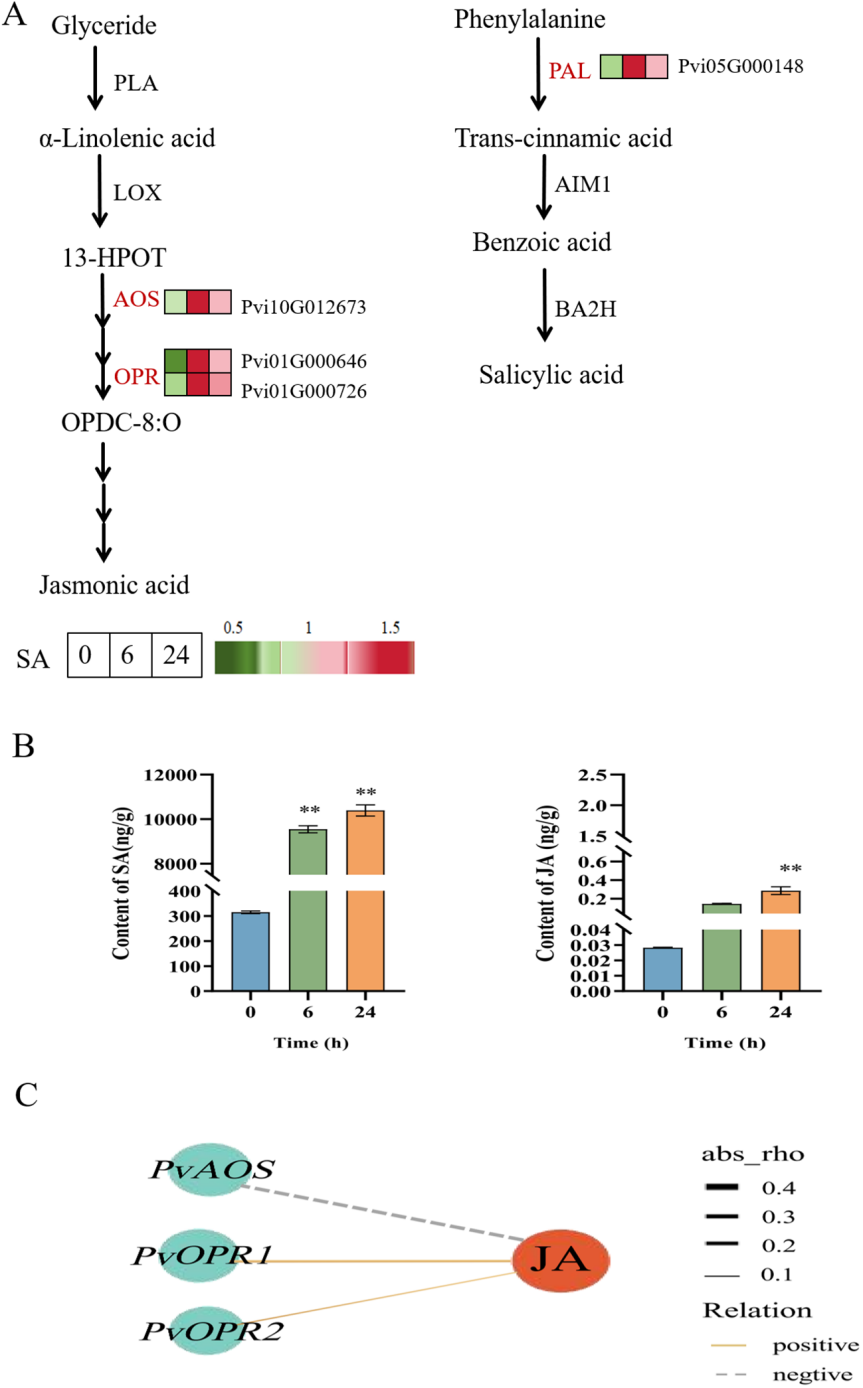
Changes of expression level and accumulation pattern of SA and JA genes in *P. vietnamensis* under SA treatment. **(A)** Transcriptome regulation of SA and JA biosynthesis and transport. The expression of DEGs is expressed by heat map, red and green represent high and low abundance. PLA, phospholipase A, LOX, Lipoxygenase, AOS, allyl oxide synthase, OPR, 12- oxygen-plant dienate reductase, PAL, phenylalanine ammonia-lyase, BA2H, benzoic acid 2-hydroxylase, AIM1, abnormal inflorescence meristem 1. **(B)** The contents of endogenous SA and JA in *P. vietnamensis* were determined after SA treatment. All the data are the average values of the three biological repeats, and the error bars represent the standard deviations of the three repeats. The asterisk indicated significant level (P < 0.05), and Tukey’s significant difference test was used. **(C)** The interaction network of DEGs and DEMs involved in JA biosynthesis. The solid line indicates positive correlation, the dotted line indicates negative correlation, the red circle indicates DAM, the blue circle indicates DEGs, and the thickness of the line indicates the correlation strength. Asterisks indicate the significant level (n = 3, **P < 0.01) based on a Tukey’s honestly significant difference test.

Combined with the results of the KEGG pathway analysis, the investigation of the genes related to SA and JA biosynthesis pathways showed that the JA synthesis-related genes (*PvAOS*, *PvOPR1*, and *PvOPR2*) and SA biosynthesis-related gene *(PvPAL*) were upregulated in the SA-6h group (**Figure 6A**). Next, we determined the effects of exogenous SA on endogenous SA and JA levels in *P. vietnamensis* leaves. Our results showed that SA and JA were highly accumulated in the SA-24h group. The SA levels in the SA-24h group were 33- and 1.1-times higher than those in the SA-0h and SA-6h groups, respectively (**Figure 6B**). Co-expression network analysis showed that JA positively correlated with *PvOPR1* (Pvi01G000646) and *PvOPR2* (Pvi01G000726; encoding 12-oxadienate reductase) (**Figure 6C**). These results showed exogenous SA application enhanced endogenous SA and JA levels in *P. vietnamensis*. Furthermore, SA and JA are synergistic in enhancing and improving the defense and disease resistance of *P. vietnamensis.*

### SA pretreatment improved the disease resistance in *P. vietnamensis*

**Figure 7:**
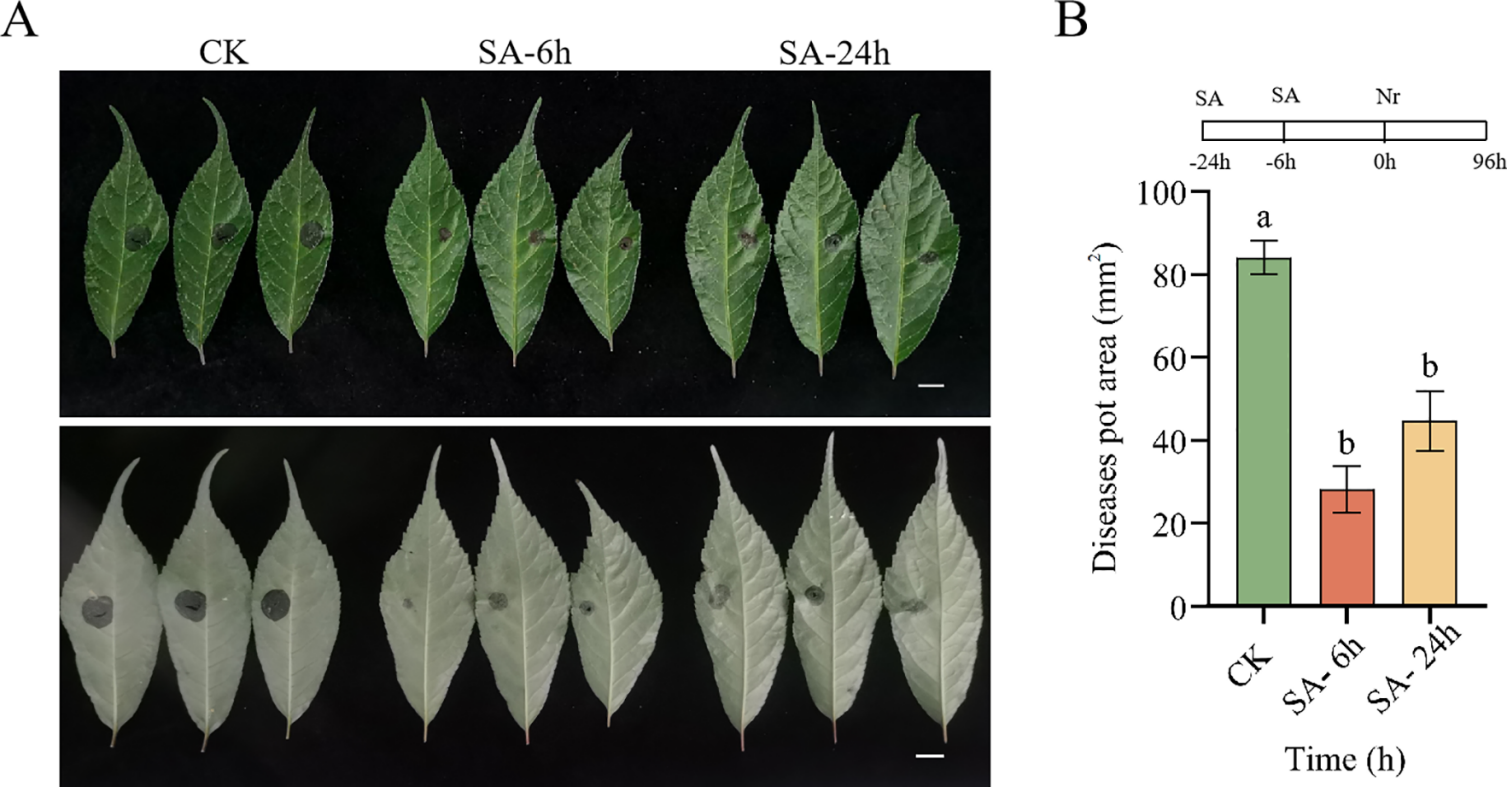
Pre-treatment with SA improved the disease resistance of *P. vietnamensis* to the fungal pathogen *Neofusicoccum ribis.* **(A)** Phenotype of *P. vietnamensis* leaves under SA pre-treatment (front and reverse side). **(B)** Lesion area size under SA pretreatment. Bar = 1 cm. The values are the means ± SEs (n = 3). Different letters indicate significant differences at P < 0.05 (Student’s t-test).

Next, to further elucidate the effects of exogenous SA application on the disease resistance of *P. vietnamensis*, we assessed the disease resistance of SA-pretreated *P. vietnamensis* against *N. ribis*. It is a fungal pathogen responsible for causing *P. vietnamensis* leaf blight. As shown in **Figure 7**, compared to the control group (CK), the SA-pretreated *P. vietnamensis* exhibited smaller leaf spots. This finding indicated that exogenous SA application can enhance the resistance of *P. vietnamensis* against *N. ribis*. This result might be attributed to SA-induced induction of defense-related gene expression and accumulation of secondary metabolites, thereby enhancing the disease resistance in *P. vietnamensis*.

## Discussion

SA, as an important plant hormone, plays a critical role in plant growth and development and stress response (Li and Li, 2019). In SA-mediated plant immunity, SA senses and mediates SA signaling via its receptor NPR1. When NPR1 binds to SA, the former translocates from the cytoplasm to the nucleus as a transcriptional co-activator, regulating transcription reprogramming and resistance to broad-spectrum pathogens (Zhang et al., 1999; Despre’s et al., 2000; Mou et al., 2003). In addition to regulating the expression of defense-associated proteins, SA also regulates the biosynthesis of resistance-related phytochemicals, a special class of secondary metabolites in plants, thus strengthening plant immunity. Studies have shown that SA positively regulates the biosynthesis of lignans/lignin via ERFli049 of the AP2/ERF transcription factor family (Ma et al., 2017). In the current study, exogenous SA application was found to enhance the accumulation of lignin and flavonoids in *P. vietnamensis* by promoting the expression of related genes (**Figure 5A**). In addition, exogenous SA treatment promoted the biosynthesis of endogenous SA and JA and the expressions of related genes (**Figures 6A, B**). These results suggested that SA mediates plant resistance by regulating the biosynthesis of *P. vietnamensis* metabolites.

To withstand diverse biotic and abiotic stresses, plants have evolved complex secondary metabolic compounds, such as phenylpropanes, terpenoids, and alkaloids. Phenylpropane metabolism is one of the most important secondary metabolic pathways in plants, producing more than 8,000 metabolites and playing an important role in the stress responses of plants (Dong and Lin, 2021). The precursor of the phenylpropane metabolic pathway is phenylalanine, which is produced via the shikimic acid pathway. Phenylalanine produces the final products via different branches of the phenylpropane metabolism pathway at all levels, such as flavonoids, hydroxycinnamates, lignin, lignans, and tannins (Gray et al., 2012). Lignin is a complex phenolic compound formed by further transportation and polymerization of the lignin monomer, which is formed via the phenylpropane metabolism pathway. The main function of lignin is to provide a structural and defensive barrier for cell walls and play an important role in plant disease resistance (Liu et al., 2018). The three basic units of natural lignin polymers are p-hydroxyphenyl (H), guaiacyl (G), and eugenol (S), which are generated by three kinds of monolignols, p-coumaryl alcohols, coniferyl alcohols, and sinapyl alcohols (Zhao, 2016). These three types of monolignols are, in turn, synthesized under the catalysis of cinnamyl alcohol dehydrogenase (CAD), caffeite/5-hydroxylate 3-O- methyl transferase (COMT), and p-coumaric acid: coenzyme A ligase (4CL). Previously, exogenous SA treatment has been reported to promote the expression of the lignin metabolic pathway-related gene (*Cs4CLb*) in tea plants, indicating that the lignin pathway participates in SA-mediated stress resistance (Liu et al., 2023). The current study showed that exogenous SA treatment promoted lignin accumulation by inducing the expressions of *PvCAD*, *PvCOMT*, and *Pv4CL* (**Figures 5A, B**). In addition, low molecular weight phenolic precursors of lignin and free radicals generated during polymerization can inactivate fungal membranes, enzymes, and toxins (Siboza et al., 2014; Singh et al., 2019). This finding was consistent with the results of the present study. Increasing POD activity and *PvPAL* expression enhances lignin content (**Figures 1B**, **5**). This cascade might be one of the disease resistance and defense mechanisms in *P. vietnamensis*.

Flavonoid metabolism is an important branch of phenylpropanoid metabolism, producing more than 6,000 kinds of polyphenols (Tohge et al., 2013). The molecular structure of flavonoids is composed of the diphenyl propane (C6‐C3‐C6) skeleton. Based on their heterocyclic C-ring, flavonoids are divided into chalcones, aurones, flavanones, flavones, isoflavones, dihydroflavonols, flavonols, leucoanthocyanidins, anthocyanidins, and flavan-3-ols (Nakayama et al., 2019). Flavonoids exhibit a high antioxidant capacity and are excellent natural antioxidants, removing excessive ROS in plants. Studies have found that anthocyanins exhibit a strong free radical scavenging activity. The three anthocyanins purified from *Solanum nigrum* exhibit several-fold higher antioxidant activity than vitamin C (Meng et al., 2020). Rutin is a flavanol and a strong antioxidant that scavenges free radicals (Wang et al., 2024). It can stop the chain reaction of free radicals, inhibit the peroxidation of polyunsaturated fatty acids on biofilm, remove lipid peroxidation products, and protect the integrity of biofilm and subcellular structure, playing an important antioxidant activity role in human skin cells. Gęgotek et al. (2019) found that the combination of ascorbic acid and rutin has a higher antioxidant activity than that of either one of them, with a strong inhibitory effect on ultraviolet rays-mediated ROS production. In addition, it can significantly increase the activity of antioxidant enzymes, activate the Nrf-2 pathway, and protect from oxidative stress. In this study, exogenous SA treatment promoted the accumulation of cyanidin and rutin and induced the expressions of *PvF3H*, *PvF3’H*, and *PvDFR* (**Figures 5A, B**). This finding showed that exogenous SA application promotes the accumulation of cyanidin and rutin, improving the disease resistance and defense ability of *P. vietnamensis*.

In addition to plant secondary metabolic pathways, SA and JA signaling pathways play an important role in plant resistance to pathogen infection (Derksen et al., 2013). The SA signaling pathway primarily regulates plant resistance to biotrophic pathogens, while the JA signaling pathway primarily mediates plant resistance to necrotrophic pathogens, herbivory, and wounding (Rajarammohan, 2021; Spanu and Panstruga, 2017; Stroud et al., 2022). SA and JA play a key role in regulating plant defense against pathogens through cross-communication signaling pathways (Pieterse et al., 2012; De Vos et al., 2005). Meanwhile, SA- and JA-mediated signaling pathways downstream of PTI and ETI also play an important role in the pathogen defense responses (Zhang et al., 2018). Many reports have described both synergistic and antagonistic effects of SA- and JA-mediated defense signaling pathways (Li et al., 2019). In the present study, we analyzed the effects of exogenous SA on the synthesis pathways of endogenous SA and JA in *P. vietnamensis*. Our results showed that SA treatment promoted the accumulation of endogenous SA and JA in *P. vietnamensis* by inducing the expressions of *PvPAL*, *PvAOS*, *PvOPR1*, and *PvOPR2* (**Figures 6A, B**). The simultaneous increase in endogenous SA and JA levels indicated a non-antagonistic coordinated interaction between SA and JA pathways during exogenous SA application. In addition, qRT-PCR results showed that the transcriptional activities of NPR1, NPR3, WRKY29, WRKY22, GTA2-1, GTA2-2, PR1, and PR5 were activated after exogenous SA treatment (**Figure 3**). Moreover, the expression level of JA-related COI1 receptors and MYC2-1 and MYC2-2 transcription factors were significantly activated after SA treatment (**Figure 3**). These results showed that exogenous SA application can increase SA and JA levels in *P. vietnamensis* and activate disease-related signaling pathways, enhancing stress resistance in *P. vietnamensis*.

## Conclusions

**Figure 8:**
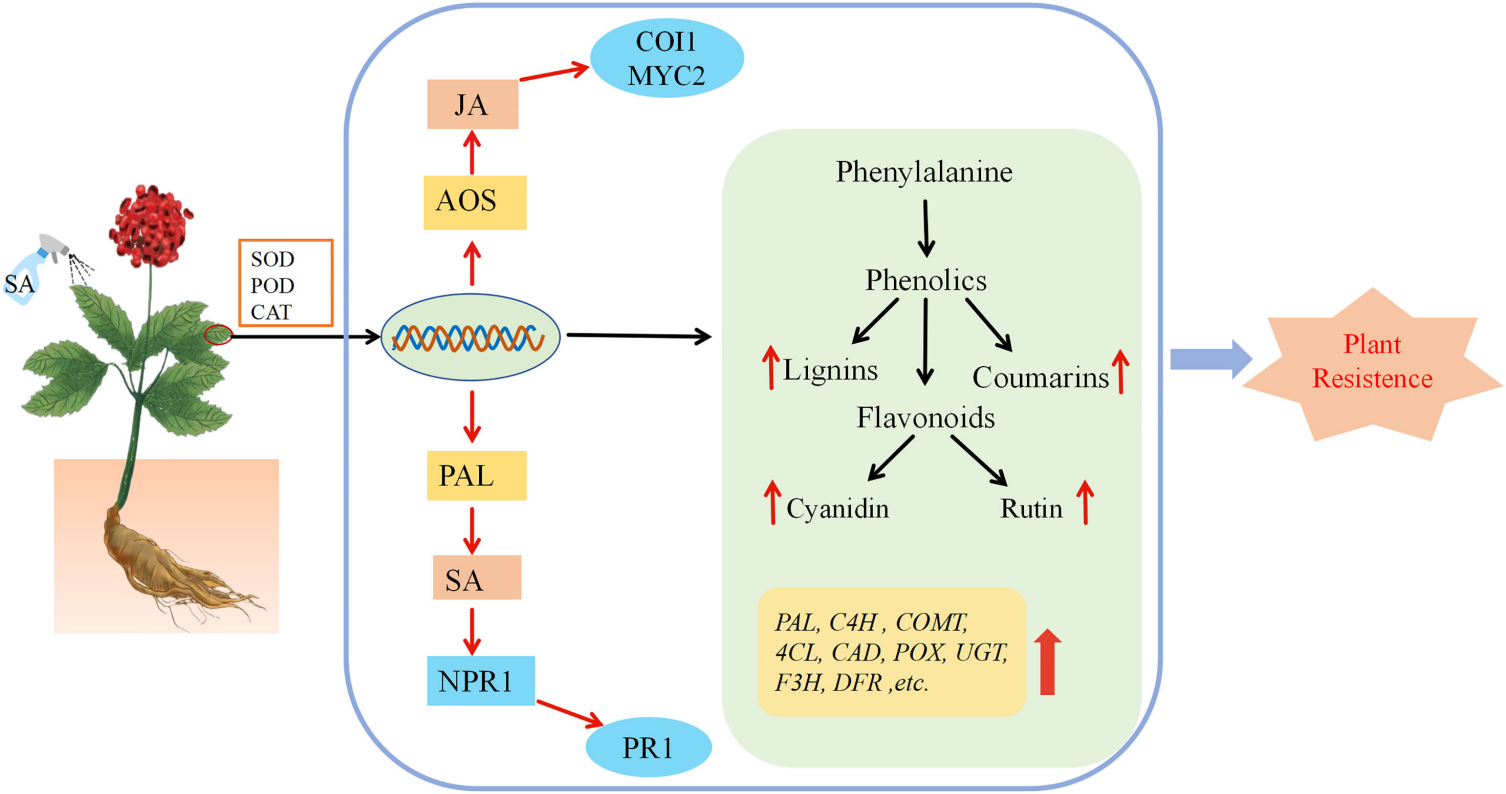
The mechanism model of the effect of exogenous SA on *P. vietnamensis*. After spraying the leaves of *P. vietnamensis* with SA, the activity of antioxidant enzymes was increased, and the transcription level changed rapidly, which eventually led to an increase in the number of important metabolites in defense-related pathways. The combined analysis of transcriptome and metabolome analysis showed that the key genes of lignin, flavonoids, salicylic acid and jasmonic acid synthesis pathway were significantly increased, resulting in the increase of lignin, rutin, cornistin, coumarin, endogenous salicylic acid and jasmonic acid, thus improving the defense and disease resistance of *P. vietnamensis*.

In this study, we investigated the impact of exogenous SA application on the biosynthesis of secondary metabolites in *P. vietnamensis* using transcriptomic and metabolomic approaches. Our results showed that lignin biosynthesis was primarily regulated by 4CL, COMT, CAD, and POX post-SA treatment. Similarly, the biosynthesis of rutin and cyanidin was primarily regulated by F3H, F3’H, and DFR. Quantitative analysis of lignin, coumarin, rutin, and cyanidin showed a marked accumulation of lignin, coumarin, and cyanidin at 24 h post-SA treatment. To the best of our knowledge, this study was the first to provide novel insights into the role of SA in the disease-resistance mechanism of *P. vietnamensis*. Overall, exogenous SA treatment promoted the biosynthesis of lignin and flavonoids in *P. vietnamensis*, enhancing its defense ability against pathogens (**Figure 8**).

## Data Availability

All sequencing dataset involved in this paper have been submitted to China National GeneBank DataBase (CNGBdb) under accession number CNP0005433.
