# Supplementary material for: Exogenous salicylic acid treatment enhances the disease resistance of Panax vietnamensis by regulating secondary metabolite production
Source: Front Plant Sci. 2024 Aug 16;15:1428272. doi: 10.3389/fpls.2024.1428272 (PMC11362055; doi:10.3389/fpls.2024.1428272)
Supplement: Supplementary file 5 [file DataSheet1.docx]

**Salicylic acid treatment enhances the disease resistance of *Panax vietnamensis* by regulating secondary metabolites**

Jiae Hou^1,2^, Mingtao Ai^1,2^, Jianbin Li^1,2^, Xiuming Cui^1,2,3,4,5^, Yuan Liu^1,2,3,4,5^ *, Qian Yang^1,2,3,4,5^ *

1 Faculty of Life Science and Technology, Kunming University of Science and Technology, Kunming 650000, China.

2 Key Laboratory of *Panax notoginseng* Resources Sustainable Development and Utilization of State Administration of Traditional Chinese Medicine, Kunming 650000, China.

3 Yunnan Provincial Key Laboratory of *Panax notoginseng*, Kunming 650000, China.

4 Kunming Key Laboratory of Sustainable Development and Utilization of Famous-Region Drug, Kunming 650000, China.

5 Sanqi Research Institute of Yunnan Province, Kunming 650000, China.

*Correspondence: Yuan Liu (liuyuan513@kust.edu.cn), Qian Yang (qian1226@vip.sina.com)

**Supplementary Information**

The supplementary information includes 2 methods, 4 tables and 3 figure.

**Supplementary Methods S1** Transcriptomic analysis

**Supplementary Methods S2** Metabolome analysis

**Supplementary Table S1** Primer information for related genes in the lignin and flavanoids synthesis pathway.

**Supplementary Table S2** List of metabolites both up-regulated and down-regulated at CK-6h vs SA-6h and CK-24h vs SA-24h.

**Supplementary Table S3** KEGG enrichment analysis of *Panax vietnamensis* leaves after 0h, 6h, 24h of SA treatment.

**Supplementary Table S4** Expression of genes involved in phenylalanine, tyrosine, tryptophan biosynthesis, and anthocyanins biosynthesis in *Panax vietnamensis* leaves SA-0h Vs SA-24h.

**Supplementary Figure S1** GO analysis on the significant down-regulated DEGs in CK-6h vs SA-6h and CK-24h vs SA-24h. Different colors represent different ontology.

**Supplementary Figure S2** Enriched KEGG pathways with significant down-regulated DEGs in CK-6h vs SA-6h and CK-24h vs SA-24h. The list of some terms KEGG pathways calculated by the Q value. The size of the dots indicates the quantity, the redder the color, the smaller the Q value. The Q value is the multiple hypothesis test-corrected P value.

**Supplementary Figure S3** Metabolic differences of CK-6h Vs SA-6h and CK-24h Vs SA-24h, green indicates up-regulated differential metabolites, orange indicates down-regulated differential metabolites.

**
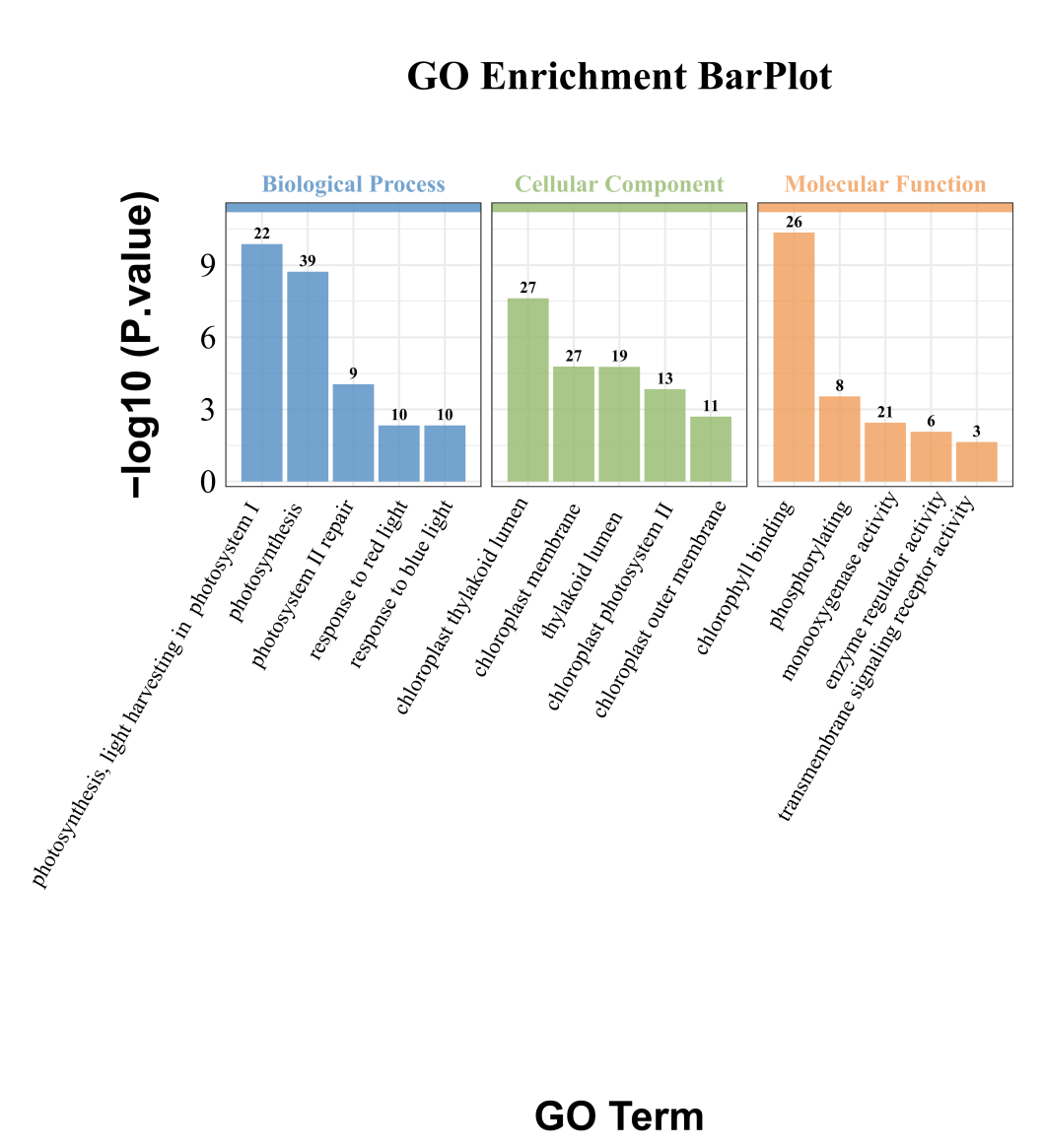
**

**Supplementary Figure S1** GO analysis on the significant down-regulated DEGs in CK-6h vs SA-6h and CK-24h vs SA-24h. Different colors represent different ontology.


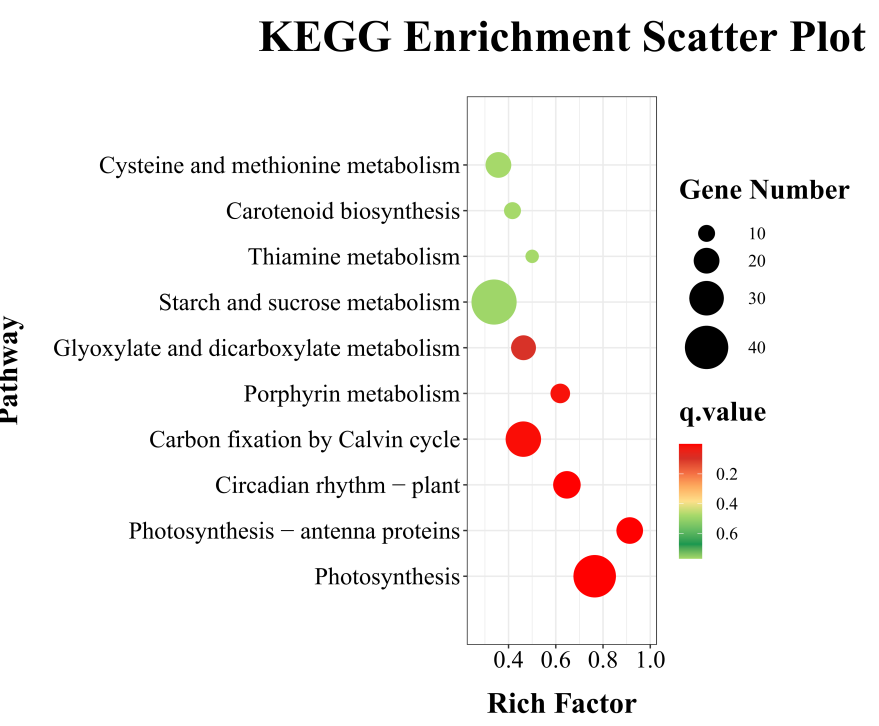


**Supplementary Figure S2** Enriched KEGG pathways with significant down-regulated DEGs in CK-6h vs SA-6h and CK-24h vs SA-24h. The list of some terms KEGG pathways calculated by the Q value. The size of the dots indicates the quantity, the redder the color, the smaller the Q value. The Q value is the multiple hypothesis test-corrected P value.


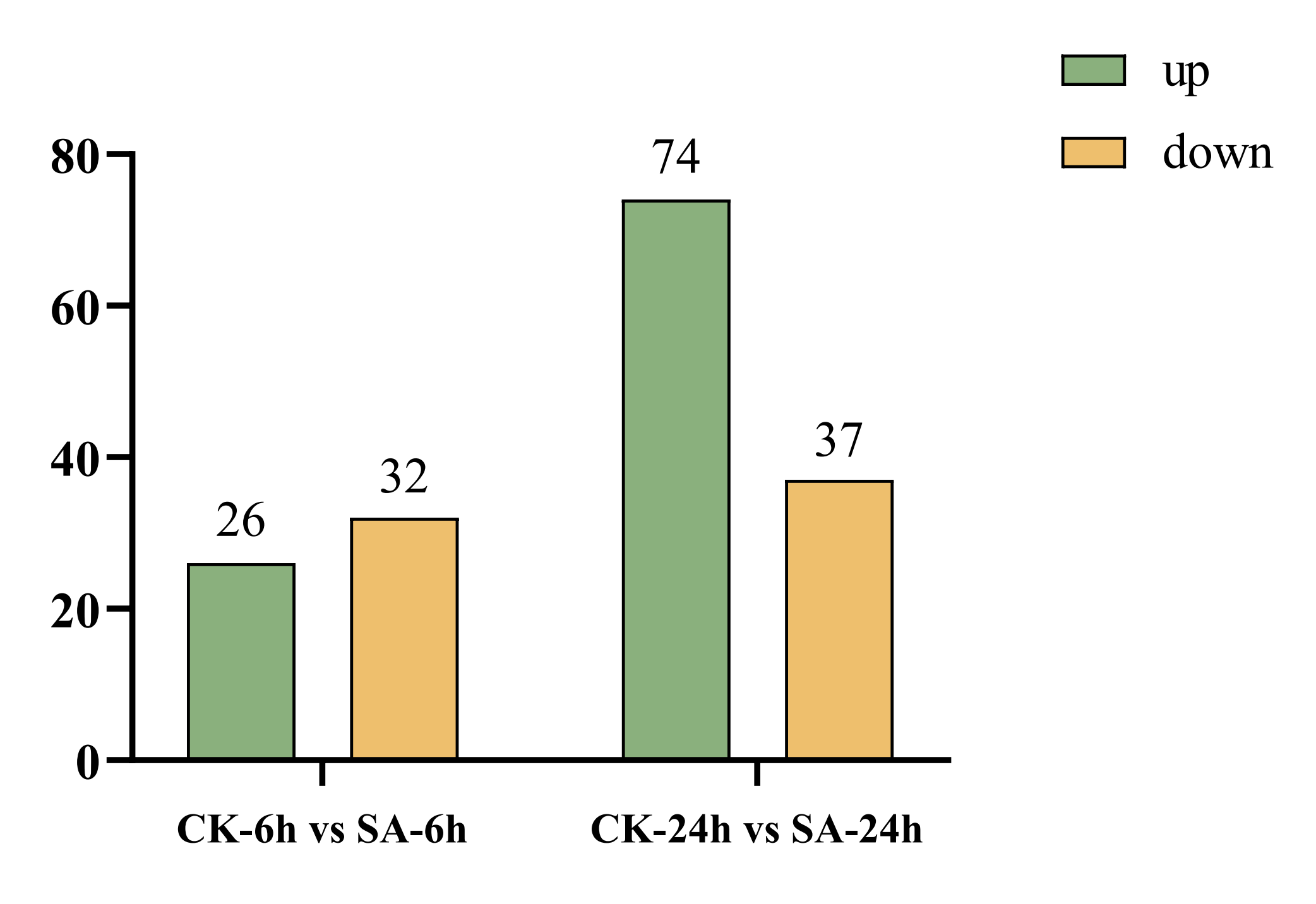


**Supplementary Figure S3** Metabolic differences of CK-6h Vs SA-6h and CK-24h Vs SA-24h, green indicates up-regulated differential metabolites, orange indicates down-regulated differential metabolites.
