# Supplementary material for: Exogenous salicylic acid treatment enhances the disease resistance of Panax vietnamensis by regulating secondary metabolite production
Source: Front Plant Sci. 2024 Aug 16;15:1428272. doi: 10.3389/fpls.2024.1428272 (PMC11362055; doi:10.3389/fpls.2024.1428272)
Supplement: Supplementary file 6 [file DataSheet2.docx]

**Supplementary Information**

The supplementary information includes 2 methods, 4 tables and 3 figure.

**Supplementary Methods S1** Transcriptomic analysis

**Supplementary Methods S2** Metabolome analysis

**Supplementary Table S1** Primer information for related genes in the lignin and flavanoids synthesis pathway.

**Supplementary Table S2** List of metabolites both up-regulated and down-regulated at CK-6h vs SA-6h and CK-24h vs SA-24h.

**Supplementary Table S3** KEGG enrichment analysis of *Panax vietnamensis* leaves after 0h, 6h, 24h of SA treatment.

**Supplementary Table S4** Expression of genes involved in phenylalanine, tyrosine, tryptophan biosynthesis, and anthocyanins biosynthesis in *Panax vietnamensis* leaves SA-0h Vs SA-24h.

**Supplementary Figure S1** GO analysis on the significant down-regulated DEGs in CK-6h vs SA-6h and CK-24h vs SA-24h. Different colors represent different ontology.

**Supplementary Figure S2** Enriched KEGG pathways with significant down-regulated DEGs in CK-6h vs SA-6h and CK-24h vs SA-24h. The list of some terms KEGG pathways calculated by the Q value. The size of the dots indicates the quantity, the redder the color, the smaller the Q value. The Q value is the multiple hypothesis test-corrected P value.

**Supplementary Figure S3** Metabolic differences of CK-6h Vs SA-6h and CK-24h Vs SA-24h, green indicates up-regulated differential metabolites, orange indicates down-regulated differential metabolites.

#### Methods S-1

**Transcriptomic analysis**

**RNA extraction library construction and sequencing**

Total RNA was extracted using Trizol reagent (thermofisher, 15596018) following the manufacturer's procedure. The total RNA quantity and purity were analysis of Bioanalyzer 2100 and RNA 6000 Nano LabChip Kit (Agilent, CA, USA, 5067-1511) , high-quality RNA samples with RIN number > 7.0 were used to construct sequencing library. After total RNA was extracted, mRNA was purified from total RNA (5ug) using Dynabeads Oligo (dT) (Thermo Fisher, CA, USA) with two rounds of purification. Following purification, the mRNA was fragmented into short fragments using divalent cations under elevated temperature (Magnesium RNA Fragmentation Module (NEB, cat.e6150, USA) under 94℃ 5-7min). Then the cleaved RNA fragments were reverse-transcribed to create the cDNA by SuperScript™ II Reverse Transcriptase (Invitrogen, cat. 1896649, USA), which were next used to synthesise U-labeled second-stranded DNAs with E. coli DNA polymerase I (NEB, cat.m0209, USA), RNase H (NEB, cat.m0297, USA) and dUTP Solution (Thermo Fisher, cat.R0133, USA). An A-base was then added to the blunt ends of each strand, preparing them for ligation to the indexed adapters. Each adapter contained a T-base overhang for ligating the adapter to the A-tailed fragmented DNA. Dual-index adapters were ligated to the fragments, and size selection was performed with AMPureXP beads. After the heat-labile UDG enzyme (NEB, cat.m0280, USA) treatment of the U-labeled second-stranded DNAs, the ligated products were amplified with PCR by the following conditions: initial denaturation at 95℃ for 3 min; 8 cycles of denaturation at 98℃ for 15 sec, annealing at 60℃ for 15 sec, and extension at 72℃ for 30 sec; and then final extension at 72℃ for 5 min. The average insert size for the final cDNA librarys were 300±50 bp. At last, we performed the 2×150bp paired-end sequencing (PE150) on an Illumina Novaseq™ 6000 (LC-Bio Technology CO., Ltd., Hangzhou, China) following the vendor's recommended protocol.

**Sequence analysis**

A cDNA library constructed by technology from the pooled RNA from < sample description > samples of < research species > was sequenced run with Illumina NovaseqTM 6000 sequence platform. Using the Illumina paired-end RNA-seq approach, we sequenced the transcriptome, generating a total of millon 2 x 150 bp paired-end reads. Reads obtained from the sequencing machines includes raw reads containing adapters or low quality bases which will affect the following assembly and analysis. Thus, to get high quality clean reads, reads were further filtered by Cutadapt (https://cutadapt.readthedocs.io/en/stable/, version:cutadapt-1.9). Then sequence quality was verified using FastQC (http://www.bioinformatics.babraham.ac.uk/projects/fastqc/, 0.11.9). including the Q20, Q30 and GC-content of the clean data.After that, a total of G bp of cleaned, paired-end reads were produced. The raw sequence data have been submitted to the NCBI Gene Expression Omnibus (GEO) datasets with accession number <GEO accession >or NCBI Short Read Archive (SRA) with accession number with accession number < SRA accession >.

**Differentially expressed genes (DEGs) Analysis**

Genes differential expression analysis was performed by DESeq2 software between two different groups (and by edgeR between two samples). The genes with the parameter of false discovery rate (FDR) below 0.05 and absolute fold change ≥ 2 were considered differentially expressed genes. Differentially expressed genes were then subjected to enrichment analysis of GO functions and KEGG pathways.

**GO Enrichment Analysis**

GO enrichment analysis provides all GO terms that significantly enriched in DEGs comparing to the genome background. Firstly all DEGs were mapped to GO terms in the Gene Ontology database (http://www.geneontology.org/), gene numbers were calculated for every term, significantly enriched GO terms in DEGs comparing to the genome background were defined by hypergeometric

test.

**Pathway Enrichment Analysis (KEGG)**

Genes usually interact with each other to play roles in certain biological functions. Pathway-based analysis helps to further understand genes biological functions. KEGG is the major public pathway-related database. Pathway enrichment analysis identified significantly enriched metabolic pathways or signal transduction pathways in DEGs comparing with the whole genome background. Pathways meeting this condition with p < 0.05 were defined as significantly enriched pathways in DEGs.

**Gene Set Enrichment Analysis (GSEA)**

We performed gene set enrichment analysis using software GSEA (v4.1.0) and MSigDB to identify whether a set of genes in specific GO terms, KEGG pathways, DO terms (for Homo sapiens), Reactome (for a few model animals) shows significant differences in two groups. Briefly, we input gene expression matrix and rank genes by Signal2Noise normalization method. Enrichment scores and p value was calculated in default parameters. GO terms, KEGG pathways

(DO terms, Reactome) meeting this condition with |NES|>1, NOM p-val<0.05, FDR q-val<0.25 were considered to be different in two groups.

**Alternative Splicing Analysis**

rMATS (version 4.1.1) (http://rnaseq-mats.sourceforge.net) was used to identify alternative splicing events and analyze differential alternative splicing events between samples. We identified AS events with a false discovery rate (FDR) < 0.05 in a comparison as significant AS events.

**Methods S-2**

**Metabolome analysis**

**Metabolite extraction**

Metabolite extraction and analysis were performed by Hangzhou Lianchuan Biotechnology Co., Ltd.The collected samples were thawed on ice, and metabolite were extracted with 80% methanol Buffer. Briefly, 50 mg of sample was extracted with 0.5 ml of precooled 80% methanol. The extraction mixture was then stored in 30 min at -20°C. After centrifugation at 20,000 g for 15min, the supernatants were transferred into new tube to and vacuum dried. The samples were redissolved with 100μL 80% methanol and stored at -80°C prior to the LC-MS analysis. Inaddition, pooled QC samples were also prepared by combining 10 μ L of each extraction mixture.All samples were acquired by the LC-MS system followed machine orders. Firstly, all chromatographic separations were performed using an UltiMate 3000 UPLC System (Thermo Fisher Scientific, Bremen, Germany). An ACQUITY UPLC T3 column (100mm*2.1mm, 1.8µm, Waters, Milford, USA) was used for the reversed phase separation. The column oven was maintained at 40°C. The fter, 5mM ammonium acetate and 5mM acetic acid) and solvent B(Acetonitrile). low rate was 0.3 ml/min and the mobile phase consisted of solvent A . Gradient elution conditions were set as follows: 0～0.8 min, 2% B; 0.8～2.8 min, 2% to 70% B; 2.8～5.6min, 70% to 90% B; 5.6~6.4 min, 90% to 100% B; 6.4~8.0 min, 100% B; 8.0～8.1 min, 100% to 2% B; 8.1～10 min, 2%B.

**Detection of metabolites**

A high-resolution tandem mass spectrometer TripleTOF 6600 (SCIEX, Framingham, MA, USA) was used to detect metabolites eluted form the column. The Q-TOF was operated in both positive and negative ion modes. The curtain gas was set 30 PSI, Ion source gas1 was set 60 PSI, Ion source gas2 was set 60 PSI, and an interface heater temperature was 500 ℃.For positive ion mode, the Ionspray voltage floating were set at 5000 V, respectively. For negative ion mode, the Ionspray voltage floating were set at -4500V, respectively. The mass spectrometry data were acquired in IDA mode. The TOF mass range was from 60 to 1200 Da. The survey scans were acquired in 150 ms and as many as 12 product ion scans were collected if exceeding a threshold of 100 counts per second (counts/s) and with a 1+ charge-state. Dynamic exclusion was set for 4 s.During the acquisition, the mass accuracy was calibrated every 20 samples. Furthermore, in order to evaluate the stability of the LC-MS during the whole acquisition, a quality control sample (Pool of all samples) was acquired after every 10 samples.

**Identification and quantification of the metabolites**

The acquired MS data pretreatments including peak picking, peak grouping, retention time correction, second peak grouping, and annotation of isotopes and adducts was performed using XCMS software. LC−MS raw data files were converted into mzXML format and then processed by the XCMS, CAMERA and metaX toolbox implemented with the R software. Each ion was identified by combining retention time (RT) and m/z data. Intensities of each peaks were recorded and a three dimensional matrix containing arbitrarily assigned peak indices (retention time-m/z pairs), sample names (observations) and ion intensity information (variables) was generated.

The online KEGG, HMDB database was used to annotate the metabolites by matching the exact molecular mass data (m/z) of samples with those from database. If a mass difference between observed and the database value was less than 10 ppm, the metabolite would be annotated and themolecular formula of metabolites would further be identified and validated by the isotopic distribution measurements. We also used a in-house fragment spectrum library of metabolites to validate the metabolite identidification.

The intensity of peak data was further preprocessed by metaX. Those features that were detected in less than 50% of QC samples or 80% of biological samples were removed, the remaining peaks with missing values were imputed with the k-nearest neighbor algorithm to further improve the data quality. PCA was performed for outlier detection and batch effects evaluation using the pre-processed dataset. Quality control-based robust LOESS signal correction was fitted to the QC data with respect to the order of injection to minimize signal intensity drift over time. In addition, the relative standard deviations of the metabolic features were calculated across all QC samples, and those > 30% were then removed.

**Differential metabolite statistics**

Univariate analysis of difference multiple (fold-change) and t.test statistical tests were used to obtain q-value and multivariate statistical analysis of VIP (Variable Important for the Projection) values obtained from PLS-DA to screen for differentially expressed metabolic ions. The difference ions simultaneously meet: ratio> = 1.5 or ratio <= 1 / 1.5; p value <0.05;. VIP ≥ 1
